# Supplementary material for: Impact of Feeder Access and Stocking Density on Tail Injuries and Performance in Weaned Piglets
Source: Animals (Basel). 2025 Jun 13;15(12):1749. doi: 10.3390/ani15121749 (PMC12189744; doi:10.3390/ani15121749)
Supplement: Supplementary file 1 [file animals-15-01749-s001.zip › animals-3624357-supplementary.pdf]

S1. Human endpoint

Humane endpoints were defined as a situation in which the piglet was clinically ill without the prospect of recovery, or in case the piglet was unable to get up or to stand upright. In case the human endpoint was reached piglets were euthanized via intracardiac injection with T61® (Intervet Nederland B.V., Boxmeer, The Netherlands) after sedation with Zoletil® (Virbac Nederland B.V., Barneveld, The Netherlands).

S2. Intervention protocol

In experiment 2 and 3 an intervention protocol was in place with the following intervention steps being defined:

1. If a tail/ear biting outbreak has taken place (i.e. with at least one piglet having a damaged or incomplete ear) an extra cotton rope was added to the pen
    - The intervention was considered successful if no fresh blood were observed (previous or new victims) in the first 3 days after the intervention.
    - In case the intervention outcome did not meet above criteria the intervention were considered not successful and we proceeded to step 2. This was done as soon as fresh blood was observed within one of the first 3 days after the intervention.
2. If intervention step 1 was not successful another enrichment material was added (i.e. a jute sack).
    - The intervention was considered successful if no fresh blood was observed (previous or new victims) in the first 3 days after the intervention.
    - In case the intervention outcome did not meet above criteria the intervention was not considered successful and we proceeded to step 3.
3. Removal of biter(s) from pen and moved to a hospital pen.

S3. Experiment 1: Stocking density

Figure S1 illustrates the prevalence of tail injuries within the different weaning rounds, experimental rooms, and replicates of experiment 1. The experiment was performed in 3 weaning rooms with in each weaning round 8 replicates, with 4 replicates per weaning room. Weaning round did not significantly influence tail injuries at day (d) 35 post-weaning (PW; P = 0.79). In weaning round 1, 96.3% of the piglets had intact tails (with 2.5% damaged tails and 1.3% incomplete tails), this was 97.5% in weaning round 2 (with 2.5% incomplete tails), and 96.2% in weaning round 3 (with 3.8% damaged tails). Ear injuries were hardly observed in this experiment (i.e. 99.6% intact and 0.4% damaged). Figure S2 illustrates the indoor and outdoor temperatures, as well as the absolute temperature differences between day and night. From those graphs it seems that during weaning round 3 the absolute temperature differences between day and night were higher (i.e. 7.27°C ± SD = 3.062) compared with weaning round 1 (i.e. 5.38 °C ± SD = 2.024) and 2 (4.88 °C ± 2.282). Between d0-14 PW the absolute temperature differences between day and night were however higher in round 1 (5.87 °C ± 2.180) than

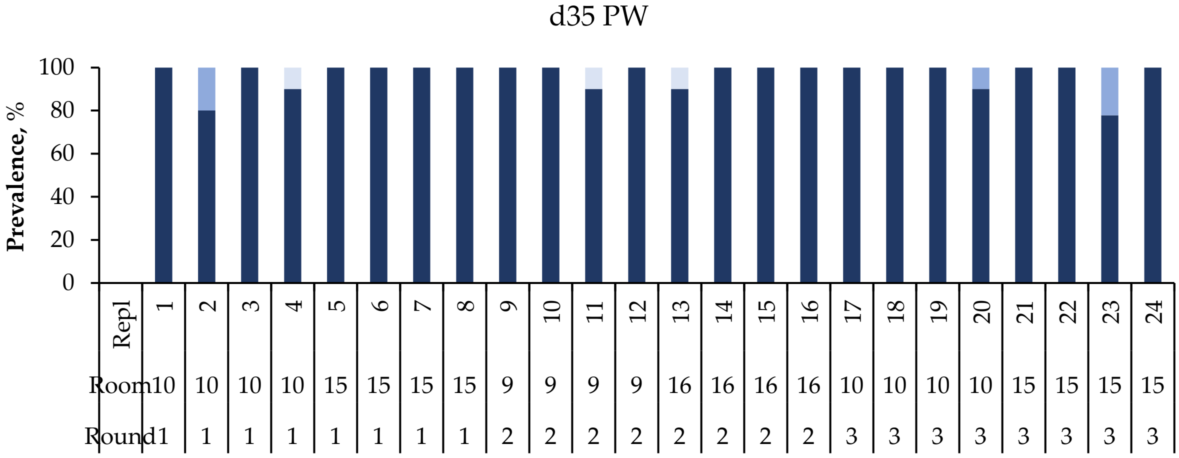

Figure S1. The prevalence of tail injuries post-weaning (PW) as expressed per weaning round, room, and replicate in experiment 1.[1]

[1] ▪ represents intact tails, ▪ represents damaged tails, and ▪ represents incomplete tails.

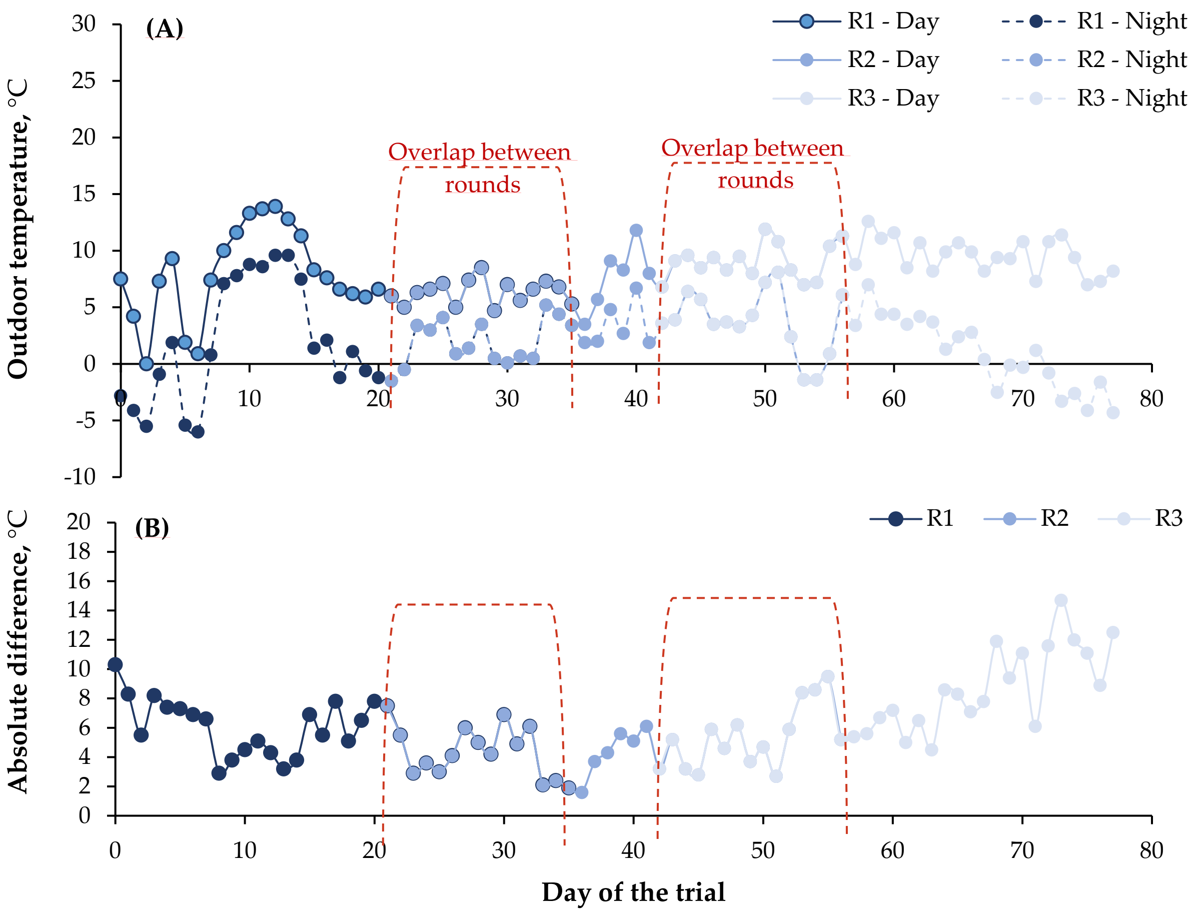

Figure S2. Outdoor temperature during day and night (A) and the absolute difference between day and night (B) during the difference weaning rounds of experiment 1 (performed winter 2021/ 2022).[1]

[1] Data came from weerstatistieken.nl.

in round 2 (4.41 °C ± 1.766) and round 3 (5.32 °C ± 2.153). Also experimental room did not significantly influence tail injuries at d35 PW (P = 0.61). Although replicate seemed to significantly influence the prevalence of tail injuries (P < 0.001), the multiple comparison did not appear to be different. This

might be due to the different methodologies used, the number of independent variables, a weak significant global effect, or for instance a conservative pairwise comparison test

S4. Experiment 2: Feeder access 1

Figure S3 illustrates the prevalence of tail injuries within the different weaning rounds, experimental rooms, and replicates of experiment 2. The experiment was performed in 2 weaning rooms with in each weaning round 6 replicates, however the experiments were in different weaning rooms. Weaning round did significantly influence tail injuries at d14 PW ($P = 0.01$) but not at d35 PW ($P = 0.33$). Tail injuries were mostly seen in weaning round 1 with 95.8% intact tails at d14 PW (4.2% damaged tails) and 89.6% intact tails at d35 PW (with 10.4% damaged tails) which was 100% and 91.7% (with 6.3% damaged tails and 2.1% incomplete tails) respectively at d14 and d35 PW for weaning round 2. Ear injuries were not observed in this experiment. Figure S4 illustrates the indoor and outdoor temperatures, as well as the absolute temperature differences between day and night. From those graphs it seems that during weaning round 1 the absolute temperature differences between day and night were higher (i.e. 5.49 °C ± SD = 2.465) compared with weaning round 2 (4.79 °C ± 2.453), though between d0-14 PW, the absolute temperature differences between day and night were higher in round 2 (5.52 °C ± 2.549) than in round 1 (4.16 °C ± 1.672).

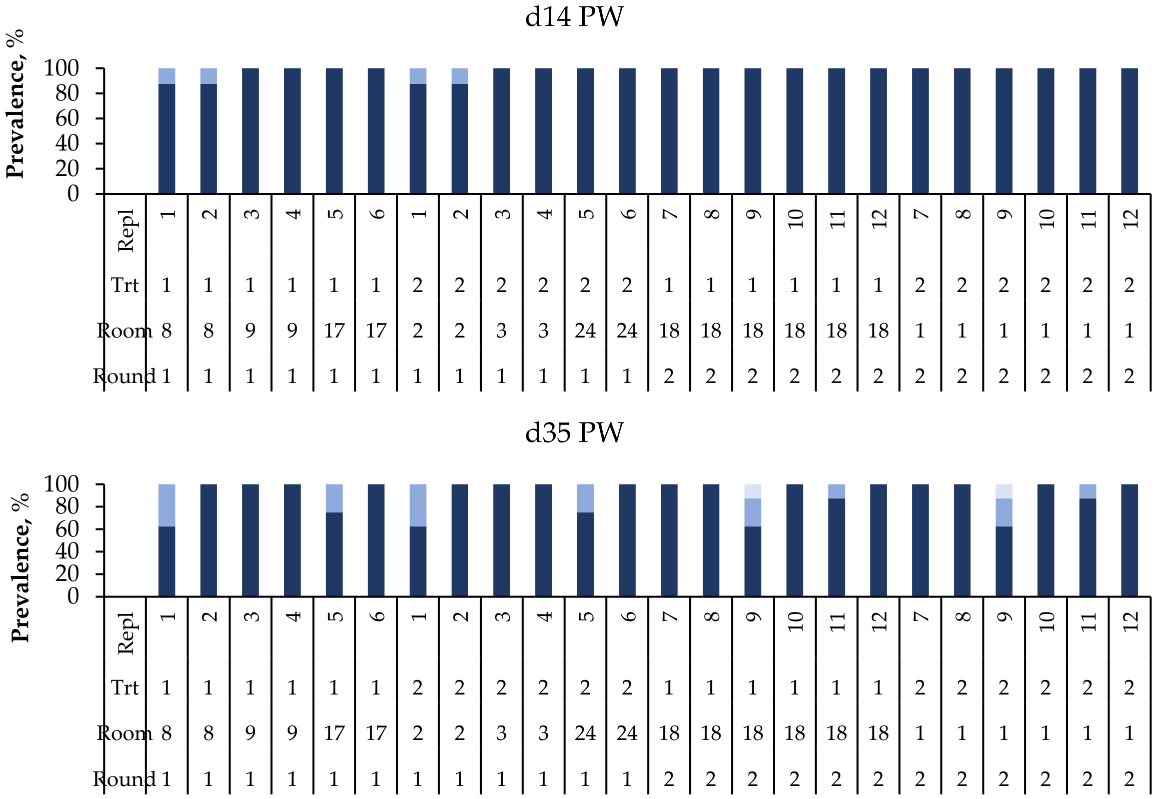

Figure S3. The prevalence of tail injuries post-weaning (PW) as expressed per weaning round, room, and replicate in experiment 2.[1]

[1] ▪ represents intact tails, ▪ represents damaged tails, and ▪ represents incomplete tails.

Replicate significantly influenced tail injuries at d35 PW ($P < 0.001$), piglets in replicate 1 (round 1) and 9 (round 2) had significant less intact tails (i.e. 62.5% intact tails and 37.5% damaged tails) compared with piglets in replicate 11 (round 2; 87.5% intact tails and 12.5% damaged tails). Replicate tended to influenced tail damage at d35 PW ($P = 0.06$), but multiple comparisons did not.

In weaning round 2 a total of 1 intervention (i.e. the addition of an extra cotton rope) was applied at d33 PW in replicate 9 of the HFA1 treatment.

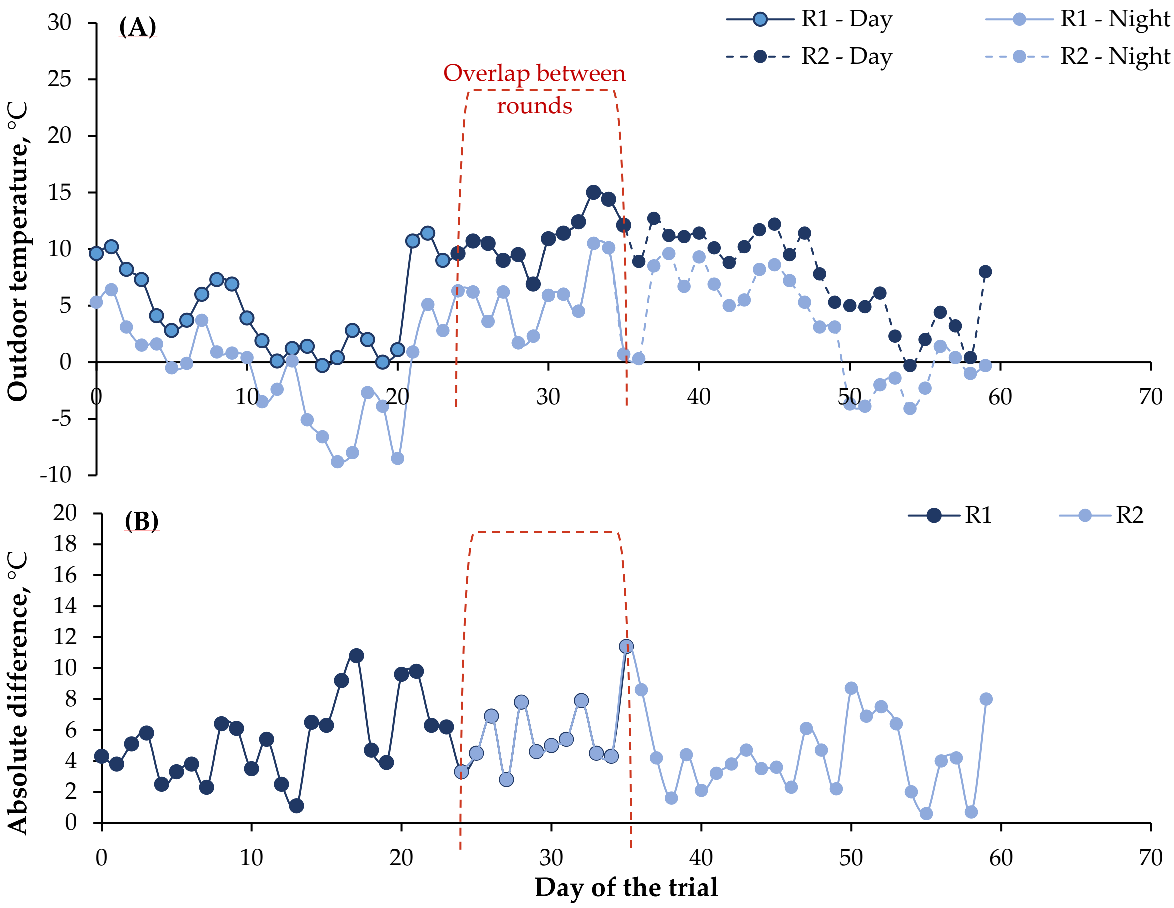

Figure S4. Outdoor temperature during day and night (A) and the absolute difference between day and night (B) during the difference weaning rounds of experiment 2 (autumn 2022 – winter 2023).[1]

[1] Data came from weerstatistieken.nl.

S5. Experiment 3: Feeder access 2

Figure S5 illustrates the prevalence of tail injuries within the different weaning rounds, experimental rooms, and replicates of experiment 3. The experiment was performed in 2 weaning rooms and the same rooms (i.e. room 21 and 22) were used in each weaning round. Weaning round significantly influenced tail injuries at d14 PW (P < 0.001) and tended to influence the prevalence of tail injuries (P = 0.08) at d35 PW. Tail injuries were mostly seen in weaning round 2 with 95.8% intact tails at d14 PW (4.2% damaged tails) and 36.7% intact tails at d35 PW (with 43.9% damaged tails and 19.4% incomplete tails) which was 100% and 52.7% (with 36.2% damaged tails and 11.1% incomplete tails) respectively at d14 and d35 PW for weaning round 1. Ear injuries were not seen in weaning round 2, but was seen in weaning round 1, but only at d14 PW (0.8% damaged ears). Figure S6 illustrates the indoor and outdoor temperatures, as well as the absolute temperature differences between day and night. From those graphs it seems that during weaning round 2 the absolute temperature differences between day and night were higher throughout the experiment (i.e. 7.31 °C ± SD = 2.703) compared with weaning round 1 (5.36 °C ± 2.587). Also between d0-14 PW, the absolute temperature differences between day and night were higher in round 2 (7.33°C ± 2.992) than in round 1 (5.07°C ± 2.374).

Experimental room tended to influence tail injuries at d14 PW (P = 0.06) with piglets reared in weaning room 21 tended to have more tail injuries than in weaning room 22 (3.3% damaged tails versus 0.8% damaged tails, respectively). Replicate significantly influenced tail injuries at d14 PW (P < 0.001), with piglets in replicate 7 (round 2, room 21) having significant less intact tails (i.e. 80.0%

intact tails and 20.0% damaged tails) compared with piglets in replicate 11 (round 2, room 22) (i.e. 95.0% intact tails and 5.0% damaged tails), whilst the rest of the replicates all had intact tails. Experimental room and replicate did not significantly influenced tail injuries at d 35 PW (P = 0.37 and P = 0.42, respectively).

In weaning round 1 a total of 1 intervention (i.e. the addition of an extra cotton rope) was applied at d30 PW for replicate 6 of the LFA2 treatment. In weaning round 2, room 21 a total of 4 interventions were applied. Replicate 8 and 9 of the LFA2 treatment required an extra cotton rope at d29 PW and a jute sack at d33 PW. In weaning room 22, a total of 2 interventions were applied including an extra cotton rope for replicate 10 of the LFA2 treatment at d28 PW and an extra jute sack in the same pen at d32 PW.

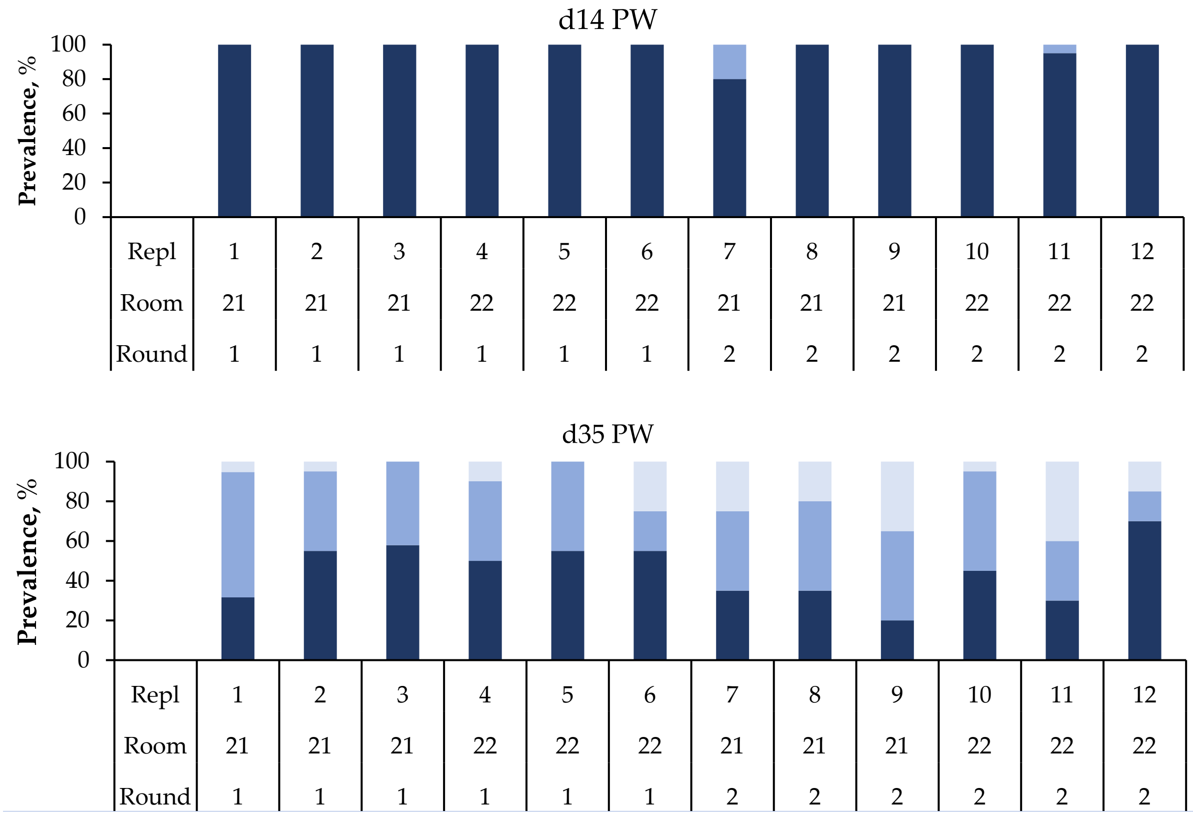

Figure S5. The prevalence of tail injuries post-weaning (PW) as expressed per weaning round, room, and replicate in experiment 3.[1]

[1] ▪ represents intact tails, ▪ represents damaged tails, and ▪ represents incomplete tails.

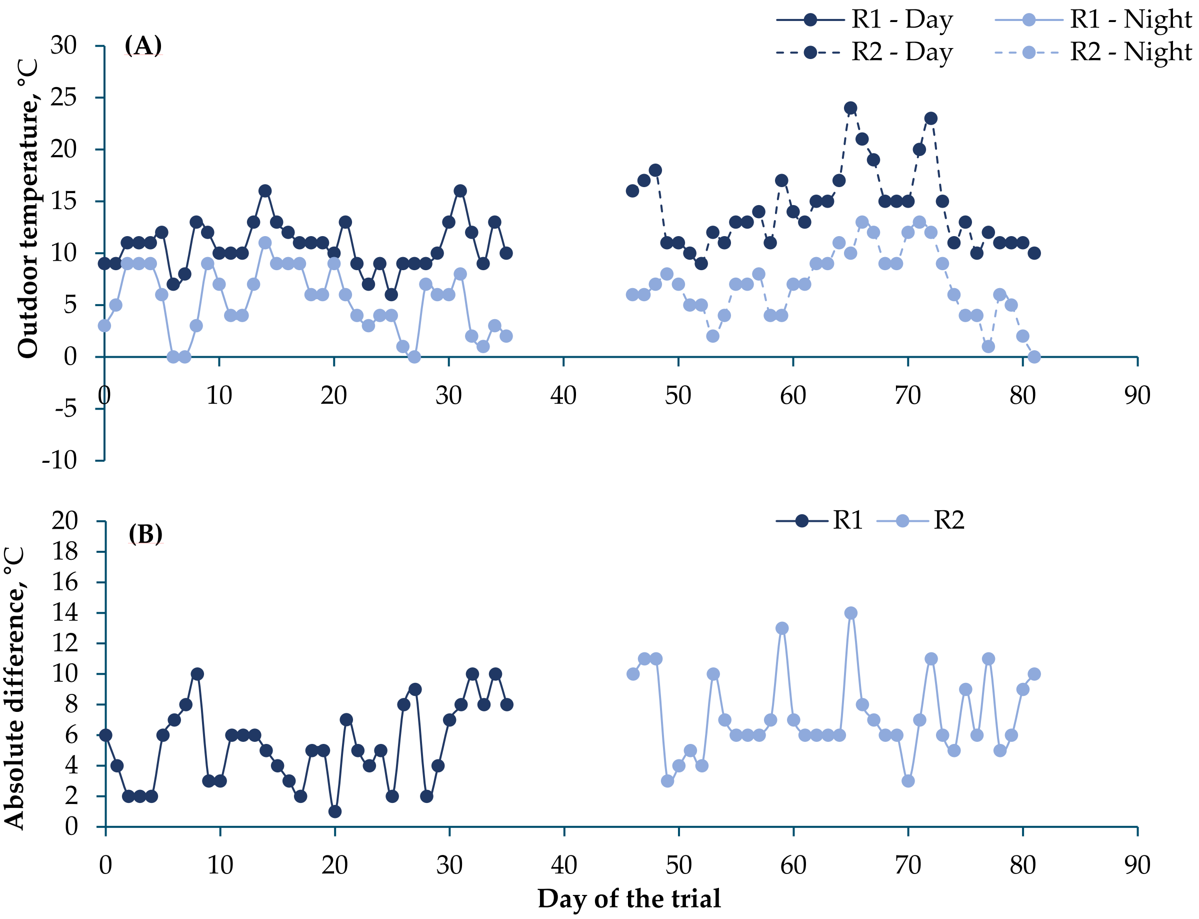

Figure S6. Outdoor temperature during day and night (A) and the absolute difference between day and night (B) during the difference weaning rounds of experiment 3 (spring 2024).[1]

[1] Data came from accuweather.com.
